# Itching Caused by TRPV3 (Transient Receptor Potential Vanilloid-3) Activator Application to Skin of Burn Patients

**DOI:** 10.3390/medicina56110560

**Published:** 2020-10-25

**Authors:** Hye One Kim, Kim Jin Cheol, Kong Yu Gyeong, Kwak In Suk

**Affiliations:** 1 Department of Dermatology, Kangnam Sacred Heart Hospital, Hallym University College of Medicine, Seoul 07441, Korea; hyonekim@gmail.com (H.O.K.); aiekfne@naver.com (K.J.C.); 2 Department of Anesthesiology and Pain Medicine, Burn Center, Hangang Sacred Heart Hospital, Hallym University College of Medicine, 94-200 Yeoungdeungpo-dong, Yeoungdeungpo-gu, Seoul 150-710, Korea; ygkong@hallym.or.kr

**Keywords:** burn scar, carvacrol, postburn pruritus, TRPV3

## Abstract

*Background and objectives:* Postburn pruritus is a common complication of scars in burn patients. In our previous study, we discovered increased expression of TRPV3, TRPV4, and TRPA1. Among them, TRPV3, in particular, is predominantly expressed in the epidermis of the tissue of pruritic burn scars. We sought to evaluate the correlation between the expression of TRPV3 activators and itching after application of TRPV3 activator carvacrol over burn scars. *Materials and Methods:* Design: This was a double-blind clinical trial with non-randomized distribution. Setting: This study was performed in a hospital setting. Patients or participants: Patients with itching of burn scars (numerical rating scale (NRS), <3; *n* = 8; Group 1 and NRS, ≥3; *n* = 16, Group 2) and healthy volunteers (*n* = 18, Group 3) were included. Interventions: The investigational drug carvacrol (100%, 75%, and 50%) and control drug (10% ethanol) were applied over the scars using IQ Ultimate™ 1 × 1 cm chamber sheets. Main variables of interest: The presence of pruritus, erythema, edema, and other skin lesions was evaluated. The verbal NRS scores were also compared. *Results:* Carvacrol induced more itching in Group 2 than in Groups 1 and 3. In Group 2, 100%, 75%, and 50% carvacrol caused significantly more itching (NRS score, 5.18 ± 3.04, 5.18 ± 3.04, and 4.93 ± 2.96, respectively) than that in Group 1 (NRS score, 1.00 ± 1.80, 1.00 ± 1.80, and 1.00 ± 1.80, respectively) and Group 3 (NRS score, 2.38 ± 1.94, 1.27 ± 1.32, respectively). *Conclusions:* The TRPV3 activator carvacrol can cause itching if applied over burn scars. This study can help understand the mechanism and prognosis of post-burn itching and contribute to the development of its treatment.

## 1. Introduction

Pruritus following a burn injury is a common and stressful complication of burn injury. Empirical antipruritic treatment often fails to achieve a satisfactory outcome, as the mechanism has not been fully elucidated. In our previous study, we evaluated the clinical and histopathological characteristics of post-burn pruritus and found an increased expression of the transient receptor potential vanilloid channel 3 (TRPV3), TRPV4, and transient receptor potential ankyrin channel 1 (TRPA1) in the skin of these patients [1]. Among these, TRPV3, in particular, is predominantly expressed on the epidermis of pruritic burn scars. A previous study reported that TRPV3 in the keratinocytes increased in scar tissue compared to normal tissue in burn patients, and thymic stromal lymphopoietin (TSLP) was activated by Keratinocytes [2]. However, the functional role of TRPV3 in keratinocytes was not clearly identified.

One of the TRP family, TRPV3, has been found in many tissues and cells, and it is related to calcium permeability. This has been known to increase TRPV3 in cardiac hypertrophic patients [3,4,5,6,7,8]. However, the role of TRPV3 in dermal fibrosis related to scar tissue is required. It is secreted from the natural herbs *Origanum vulgare* and *Thymus vulgaris,* such as carvacrol [2-methyl-5-(1-methylethyl) phenol]; it is a monoterpene phenol extract; and it possesses a variety of biological and pharmacological activities, such as hepatoprotective, analgesic, antitumor, antioxidant, and anti-inflammatory properties. It is also a potent activator of TRPV3, and is a known mediator [4]. Chronic inflammatory diseases and skin aging is known to be useful as a treatment. The mechanism of the collagen gene was also discovered [4,6]. The TRPV3 is important to reveal the itching of burn scars. The purpose of this study was to determine whether the TRPV3 activator carvacrol causes pruritus and its topical action on burn scars.

## 2. Materials and Methods

### 2.1. Reagents

Carvacrol was purchased from Sigma Aldrich (St. Louis, MO, USA) and was dissolved in ethanol.

### 2.2. Patient Selection

Patients with itching of burn scars (NRS (numerical rating scale), <3; *n* = 8; Group 1 and NRS, ≥ 3; *n* = 16, Group 2) and healthy volunteers without any history of burn injury (*n* = 18, Group 3) were included. The patients visited Hangang Sacred Heart Hospital, Hallym University, Seoul, Korea.

Written informed consent was obtained from each patient before surgery. The study was approved by the Bioethics Committee of Hangang Sacred Heart Hospital of Hallym University (No. 2019-035, 12 February 2020). The Institutional Review Board of Hangang Sacred Heart Hospital approved the study protocol. Written informed consent was obtained from each patient or their parents/legal representatives and participants. The inclusion criteria were patients with burn injury who were ≥7 years of age at the time of examination, those who were ≥19 years of age, and those who provided written consent prior to participation in the study and who were able to follow the requirements of the clinical trial (Groups 1 and 2), as well as healthy individuals without any acute or chronic physical conditions, including skin disorders (Group 3). The exclusion criteria were those with infectious skin conditions, pregnant or breastfeeding women, patients with abnormal skin findings (infection, erythema, or telangiectasia around the site of testing), and other individuals who were determined by the principal investigator to be unfit for the study.

### 2.3. Experimental Method

**Figure 1:**
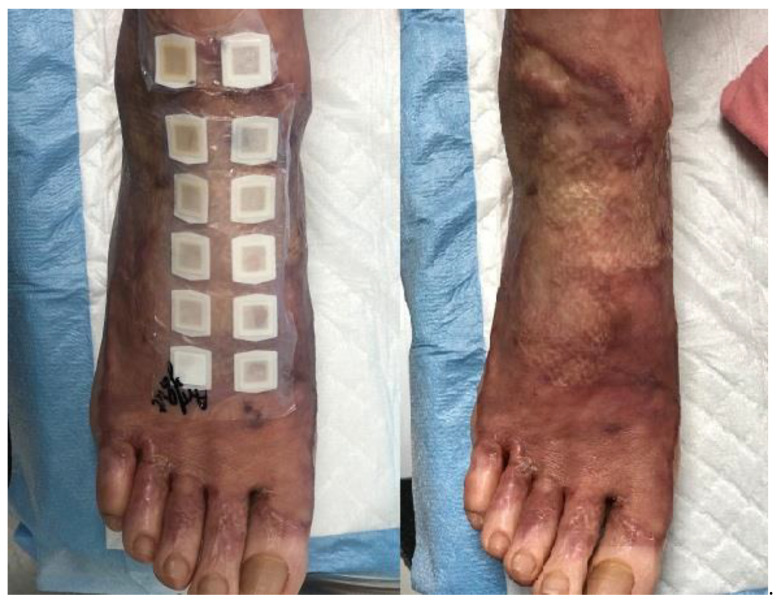
Five μL of the test drug (TRPV3 activator) and control drug (10% ethanol) soaked into each IQ Ultimate™ 1 × 1 cm sheet, and applied to the skin. The investigator evaluated whether there was any itching of the skin lesions.

This was a double-blind study. Five microliters of the investigational drug (carvacrol (100%, 75%, and 50%)) and the control drug (10% ethanol) were loaded onto IQ Ultimate™ 1 × 1 cm chamber sheets (Ecoderm Co., Sweden) and subsequently applied over the skin (Figure 1). In Groups 1 and 2, the site of testing was the burn scar lesion. In Group 3, carvacrol was applied to one upper arm (randomly chosen), and 10% ethanol was applied to the other arm. The 75% carvacrol was made by mixing 0.25 cc ethanol with 100% carvacrol of 0.75 cc per a 1 cc syringe. The 50% carvacrol was made by mixing 0.5 cc ethanol with 100% carvacrol of 0.5 cc per a 1 cc syringe.

**Figure 2:**
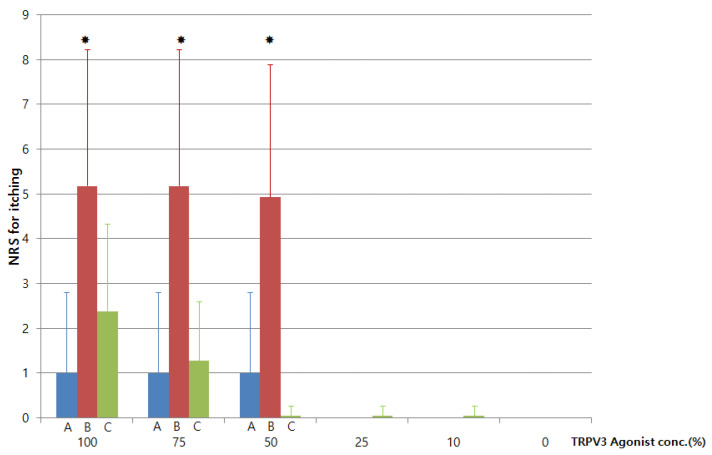
Comparative effect of carvacrol on itching among the three groups. A: Group 1 patients with no itching of the burn scars (NRS, <3; *n* = 8), B: Group 2 patients with itching of the burn scars (NRS, ≥3; *n* = 16), and C: Group 3 volunteers with burns (*n* = 18) (NRS: numerical rating scale). The values represent the mean ± SD. * *p* < 0.05.

The presence of pruritus, erythema, edema, and other skin lesions was evaluated by the investigator. The efficacy was evaluated immediately after removing the IQ Ultimate™ chamber sheets. The verbal NRS scores were recorded and classified from score 0 (no itching) to 10 (severe itching) (Figure 2). It was evaluated immediately after application and 30 min after.

### 2.4. Statistical Analyses

All data are represented as the mean ± SD. Although the initial plan was to use one-way analysis of variance, the variables were not normally distributed. Therefore, the Kruskal-Wallis test was used for statistical analysis. A *p*-value of < 0.05 was considered statistically significant. All analyses were performed using SPSS version 12.0 for Windows (SPSS Korea, Seoul, Korea).

## 3. Results

Age, gender, total body surface area (%), site, and time after burn injury were compared among the three groups (*p* < 0.001) (Table 1). The 24 burn patients consisted of Group 1 (NRS < 3, age 39.0 ± 12.6 male% 37.5, TBSA% 26.7 ± 25.5, Time after burn injury, months 83.27 ± 11.1) and Group 2 (NRS > 3, age 42.1 ± 16.2 male %18.6, TBSA% 22.0 ± 14.0, time after burn injury, months 91.43 ± 23.5). The 18 healthy people consisted of age 31.16 ± 9.2, male% 50. There was no significant difference in terms of age, gender, total body surface area, and time after burn injury among the three groups. Patients in Group 2 experienced more itching than those in Groups 1 and 3. There was no difference between nonpruritic burn scars and healthy people at the score of NRS for itching after application of carvacrol (Figure 2).

In Group 1, the change in the NRS score after the application of ethanol and carvacrol was not significant. As compared to Group 2, the administration of carvacrol (100%, 75%, and 50%) caused less itching (NRS scores, 1.00 ± 1.80, 1.00 ± 1.80, and 1.00 ± 1.80, respectively). Carvacrol induced more itching in Group 2 than in Groups 1 and 3. In Group 2, 100%, 75%, and 50% carvacrol caused significantly more itching (NRS score, 5.18 ± 3.04, 5.18 ± 3.04, and 4.93 ± 2.96, respectively) as compared to Groups 1 (NRS score, 1.00 ± 1.80, 1.00 ± 1.80, and 1.00 ± 1.80, respectively) and 3 (NRS score, 2.38 ± 1.94, 1.27 ± 1.32, respectively).

## 4. Discussion

In this study, we found that the TRPV3 activator carvacrol induced itching when applied over burn scars. TRPV3 on keratinocytes appeared mainly in the skin, esophagus, distal colon, and cornea [3,9,10,11]. In our previous study, TRPV3 was found to facilitate TSLP release from keratinocytes of burn scar tissues with itching [2]. A recent study demonstrated that carvacrol plays an important role in regulating cell migration. These results suggest that the excessive regulation of the TRPV3 channel in wound healing can lead to hypertrophic scars, representing a novel pathologic mechanism. Furthermore, because TRPV3 is also expressed on skin, the regulation of this channel may reduce scar formation with itching [12].

Pruritus following a burn injury is a common and stressful complication [13]. The reported incidence of severe itching is as high as 87% in adults and 100% in pediatric patients with burn injury [14,15]. This can greatly reduce the quality of life of patients. Post-burn pruritis usually appears two weeks after burn injuries, and may last for several months to years. Studies conducted in other countries have reported that persistent post-burn pruritis is very frequent in 87% of patients with large burns (approximately involving 19% of the body surface area) and in 35% of patients with smaller burns (approximately involving 2% of the body surface area). A Korean study conducted in 51 patients with burn injuries reported that pruritus was the most common cause of post-burn paresthesia, and most patients with large burns reported pruritus [16,17].

Post-burn pruritis may not improve with time, and may worsen. The incidence of post-burn hyperplastic scarring can be lowered through the use of skin grafts, and post-burn pain can be reduced by the use of various anti-inflammatory analgesics. In contrast, post-burn pruritis is often difficult to treat. Since it is difficult to manage post-burn pruritis by the use of general anti-histamines (H1 or H2 blockers), local anesthetics, anticonvulsants (gabapentin), antidepressants, transcutaneous electrical nerve stimulation, massage, or cognitive relaxation therapy have been attempted, but without satisfactory results. In some patients, pruritus improves only with intra-dermal steroid injections or long-term steroid use. However, patients report much more pain when drugs are injected into burn scars, as compared to that felt after injection into normal skin. Furthermore, chronic steroid use is likely to cause systemic adverse effects [18]. Despite these measures, post-burn pruritis often gets chronic and is overlooked more often as compared to other complications. Although many clinical trials on post-burn pruritis have been conducted, basic studies elucidating the mechanism of pruritis are lacking, both in Korea and other countries.

**Figure 3:**
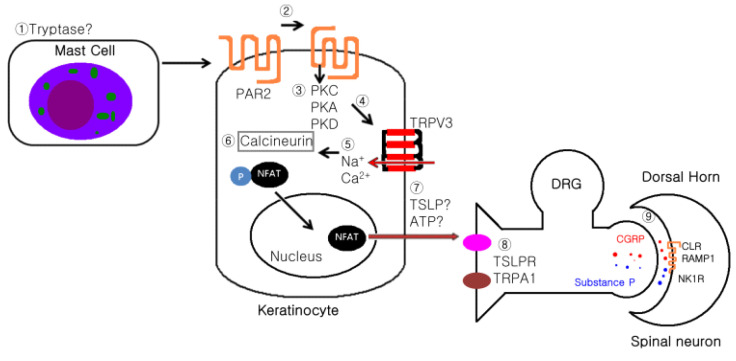
Hypothesis showing the mechanism by which pruritis is transmitted from keratinocytes to the sensory nerves.

Figure 3 shows the hypothesized mechanism through which pruritus is transmitted from keratinocytes to nerve endings. Adipocytes secrete tryptase, which binds to PAR2 in keratinocytes and activates a second messenger system in the cells. This signal sensitizes TRPV3 channels, and calcium enters the cell continuously. Calcium activates the calcium-calmodulin signaling pathway to increase the expression of TSLP. After TSLP is secreted in the keratinocytes, nerve fibers expressing TSLP receptors are activated and transmit pruritus signals to the dorsal root ganglion of the spinal cord [3,19].

In a previous experimental study that isolated the keratinocytes from burn patients’ scars with pruritis, protein kinases A (PKA) and C (PKC) were inhibited, and TRPV3 function was decreased. Expressions of TSLP mRNA and protein, and TSLPR protein increased, and TRPV3 function increased. It also showed an increase in intracellular calcium levels [2].

The present study aimed to evaluate the expression of various TRP channels in burn scars. The expression was compared to that in normal skin to investigate the correlation between TRP channel expression and pruritus. In our previous study, the expression of TRPV 1, 3, and 4 and TRPA 1 was quantified in post-burn pruritic skin, and its relationship with the severity of pruritis and burns was analyzed. Moreover, the expression of TRP channels was estimated at the protein and ribonucleic acid levels, and the correlation between each variable was investigated. The present study aimed to confirm the findings of previous reports that the expression of TRPV3 and TRPA1 highly correlates with post-burn pruritis. Research on the role of TRP channels in mediating pruritus in burn scars and skin lesions would be important in elucidating the mechanism of pruritus.

Although keratinocytes express various receptors (NMDA, GABA receptors, and TRP channels) in neurons, unlike neurons, they cannot produce action potentials. Therefore, the specific roles of these receptors should be investigated. In particular, how keratinocytes directly read and transmit the information about pruritus to the neurons requires further research. Interestingly, intra-epidermal nerve fibers are less dense in severe pruritic lesions than that in normal skin, raising the possibility that factors other than nerve fibers may play crucial roles in transmitting pruritis (Figure 3). Recent studies have suggested that keratinocytes play important roles in pruritus. Evidence supporting this finding is that itching is absent in tissues other than skin and mucosa, and pain, rather than itching, is present when the epidermis is absent due to skin injuries. While itching starts after the epidermis recovers, substances secreted by keratinocytes directly induce action potentials in neurons without the action of immune cells [5].

The TRP channels are an ion channel group located on the plasma membrane of various types of cells. It has been shown that certain thermosensitive TRP channels, especially TRPV1, TRPV3, TRPV4, and TRPA1, play important roles in the pathogenesis of pruritus and pain [20]. In a few earlier studies, the absence of TRPV3 in the TRP channels of skin cells was reported, but this finding was later contradicted in other studies [10,21]. Our results showed that in the presence of severe itching, the response to TRPV3 increases. Therefore, itching has an association with TRPV3 in burn scars.

The results of this study are subject to some limitations. First, the sample size was small. Second, since a patch-based drug delivery system was used, it could have affected the absorption of the test drug depending on the thickness and condition of the burn area. Although the prick test is more accurate, it is invasive. Third, since NRS is a subjective evaluation, it could have been affected by several factors.

## 5. Conclusions

This study showed that carvacrol, a TRPV3 activator, induced pruritis when applied over burn scar lesions. Therefore, the modulation of TRPV3 could be a possible therapeutic target for pruritis in patients with burn injuries.

## Figures and Tables

**Table 1:** The clinical differences for groups 1, 2, and 3.

	Group 1 (*n* = 8)	Group 2 (*n* = 16)	Group 3 (*n* = 18)	*p*-Value
Age	39.0 ± 12.6	42.1 ± 16.2	31.16 ± 9.2	0.077
Gender (male)%	37.5	18.6	50	0.637
Male vs Female	3/5	3/13	9/9	
TBSA (%)	26.7 ± 25.5	22.0 ± 14.0	NA	
Time after burn injury, months	83.27 ± 11.1	91.43 ± 23.5	NA	0.188

All data shown are shown as means ± SD. Group 1 of patients with no itching in burn scars (NRS < 3), Group 2 patients with itching (NRS > 3), Group 3 patients unrelated to burns (*n* = 18).
